# Conceptualising the value of simulation modelling for public engagement with policy: a critical literature review

**DOI:** 10.1186/s12961-023-01069-4

**Published:** 2023-11-27

**Authors:** Victoria Loblay, Louise Freebairn, Jo-An Occhipinti

**Affiliations:** 1 The Australian Prevention Partnership Centre, Sydney, Australia; 2 Youth Mental Health and Technology Team, Brain and Mind Centre, Faculty of Medicine and Health, The University of Sydney, Sydney, NSW Australia; 3 Menzies Centre for Health Policy and Economics, Faculty of Medicine and Health, The University of Sydney, Sydney, NSW Australia; 4 Computer Simulation & Advanced Research Technologies (CSART), Sydney, NSW Australia

**Keywords:** Participatory modelling, Complex systems, Public policy, Knowledge generation, Community engagement, Communication, Transparency, Transformational change, Multiplicity, Policymaking

## Abstract

As we face complex and dynamically changing public health and environmental challenges, simulation modelling has come to occupy an increasingly central role in public engagements with policy. Shifts are occurring not only in terms of wider public understandings of modelling, but also in how the value of modelling is conceptualised within scientific modelling communities. We undertook a critical literature review to synthesise the underlying epistemic, theoretical and methodological assumptions about the role and value of simulation modelling within the literature across a range of fields (e.g., health, social science and environmental management) that engage with participatory modelling approaches. We identified four cross-cutting narrative conceptualisations of the value of modelling across different research traditions: (1) models simulate and help solve complex problems; (2) models as tools for community engagement; (3) models as tools for consensus building; (4) models as volatile technologies that generate social effects. Exploring how these ideas of ‘value’ overlap and what they offer one another has implications for how participatory simulation modelling approaches are designed, evaluated and communicated to diverse audiences. Deeper appreciation of the conditions under which simulation modelling can catalyse multiple social effects is recommended.

**Supplementary Information:**

The online version contains supplementary material available at 10.1186/s12961-023-01069-4.

## Background

The COVID-19 pandemic has placed simulation models and modelling practice in the spotlight. Diverse publics are increasingly engaging with simulation modelling on issues ranging from health to the environment, and demands for transparency and access to model results are growing. The purpose of models and how they are made useful are now pressing topics of both scientific discussion [1, 2] and public conversation [3]. This has also prompted calls for greater transparency and scrutiny of models [4] and burgeoning commentary on ‘best practices’ for responsible, transparent modelling to ensure that these tools serve society’s best interests [5–7].

Subsequently, simulation models and their communication have been transforming. In the early days of the COVID pandemic, as governments and publics tried to make sense of the new virus, ‘Flatten the Curve’ was introduced alongside hand-washing as a key public health message. Flattening or bending the curve became a familiar visual, representing modelled projections of the effects of slowing the spread of an infectious disease outbreak. By April 2020, Forbes magazine declared ‘Flatten the curve’ an historic disease image [8]. A form of societal activism coalesced around modelling, for example, citizen scientists developed models as decision aids for office managers wondering whether and when they should send employees home [9]. With the availability of COVID vaccines in 2021, public interest in modelling turned to the question of how to safely open up alongside vaccine rollouts. Modelling practice responded to the escalating public attention by exploring new avenues for making model results publicly accessible [10]. Meanwhile, questions began to emerge around whether we ought to have other ways of framing and narrating the COVID crisis beyond the ‘obscure predicates and designs’ of modelling [11]. In the midst of such questions and efforts toward transparency, however, broader public understandings and interpretations of models and modelling practice remained somewhat unclear.

The terms 'model' and 'modelling' are used to refer to a wide range of practices including statistical modelling, systems modelling, microsimulation and economic modelling, and modelling techniques of data science. At times, the term ‘model’ may simply be used as shorthand for something that is perceived as an ideal example of a phenomenon, for example, a policy or desirable behaviour [12]. In this article, we do not focus on any particular type of model or modelling practice, however, we are interested in the broad category of simulation models aimed at forecasting future trajectories and exploring potential impacts of policy options. Simulation modelling in this context includes analytic methods of complex systems science such as system dynamics modelling, agent-based modelling, and discrete event simulation. These methods have been widely applied to sectors such as engineering, economics, defence, ecology, and business since the mid-1950s, and more recently to healthcare and population health [13–18]. Particularly, we are interested in how these models are conceptualised in relation to networks of social relations that are harnessed to respond to future national and global challenges.

The social dynamics of modelling are explored in detail in the field of participatory modelling. Participatory approaches to modelling practice are varied, but research in this area tends to be oriented toward the engagement of stakeholders and community members in modelling practice [19]. Emphasis is placed on effective communication between modellers and stakeholders, as well as among diverse stakeholder groups [20]. Understanding how core participants—as the ‘first audience’ of a model—engage with and understand models may provide clues as to how models are communicated and used with broader audiences. Policymakers engaged in participatory modelling, for example, may require models to provide concise advice accompanied by “a plausible narrative” that is relevant to areas of immediate policy concern [7]. The value of modelling is often located in participatory engagements with the model: participants are able to appreciate complexity of issues, understand the crucial role of data availability, recognise the range of influences at play when contemplating policy and planning decisions [21], or confront sensitive issues with other policy experts [22]. Much of this research investigating the value of participatory modelling for stakeholders tends to be based on case studies of modelling in particular sectors such as public health, or environmental science [23, 24]. As illustrated by the multi-dimensional environmental, socio-psychological, economic and political impacts of the COVID pandemic, however, crises, and potential solutions, are rarely confined to single disciplines or sectors [7].

Recent critical approaches to participatory modelling within science and technology studies have begun to consider how health modelling could be informed by approaches in other sectors which explore how scientists and lay publics can more effectively collaborate [25, 26]. Yet there is generally limited guidance about what learnings from different research traditions have to offer one another, or how issues of transparency and accessibility can travel beyond the core participants involved in modelling projects. In cases where models circulate with lay publics or decision-makers who have *not* been intimately involved in participatory modelling—as happened with COVID models and continues to happen with climate change modelling—the ramifications of these broader engagements and the value models hold in wider societal contexts remains unclear. To generate a deeper understanding of how models and model findings are made valuable through their circulation and socialisation with diverse publics, we undertook a critical literature review. The review covers applications of simulation modelling across systems science, environmental, biological, health systems, as well as the social sciences where critical perspectives and discussions on public engagements with modelling have been most developed. The review explores (1) How the role and value of models is conceptualised in relation to knowledge sharing, community participation and public engagement (2) The epistemic, theoretical and methodological traditions and assumptions that underpin these approaches to modelling practice. We go on to consider how critical reflections on ‘value’ from different traditions might inform the development of research on modelling practice and the judicious use of models going forward.

## Methods

As we were interested in exploring underlying assumptions within the empirical stories of the literature [27], we adopted hermeneutics as the philosophy and methodology for conducting the review [28]. Many reviews of participatory modelling focus on the question of ‘what works?’ or synthesise learnings from a particular field (i.e., health policy or environmental science). In our critical literature review, the emphasis is on induction, interpretation and critique [29], with the goal of deepening understanding of how ‘value’ is understood in different approaches to participatory modelling. Conventional systematic review methodology—testing theories around ‘what works’ through exhaustive searches, or determining averages through quantitative data—would be inappropriate for our aims [29]. Although numerous texts in the simulation modelling literature draw on insights derived from quantitative models, our critical analysis centres on unpacking the implicit assumptions conveyed through the narrative descriptions of the purpose of models and why they are valuable.

Greenhalgh and colleagues’ [30] emphasis on mapping storylines of different research traditions influenced our approach. However, we decided it was more appropriate and informative to pursue a “dynamic, recursive and reflexive” synthesis [27] of how the modelling literature conceptualised the value of models in relation to knowledge sharing and public engagement, rather than a highly systematic, meta-narrative analysis. Drawing on our collective expertise in participatory modelling and social studies of science, we prioritised texts that were relevant to highlight distinctive contributions and major themes, instead of presenting an exhaustive overview of the entire body of literature and creating strict boundaries around inclusion and exclusion criteria.

Through an iterative approach, a search strategy began with a preliminary overview of a broad range of modelling literature across different sectors to help identify key search terms and refine our research questions. After trialling searches in a number of electronic databases including Google Scholar, Web of Science, PubMed, Science Direct, and Proquest we found that Scopus enabled us to locate the most relevant papers in relation to our areas of focus. The review was conducted in two phases between August and December 2021. Sources included in the search were scholarly documents (peer reviewed journals, conference proceedings and PhD dissertations) published in English between 2006 and 2021. Initially review search terms included ‘systems model’ OR ‘systems modeling’ OR ‘systems modelling’ OR ‘systems models’ AND engagement OR engaging OR ‘policy maker’ OR ‘policy makers’ OR ‘policy makes’ yielding 890 documents. Based on themes and terms identified in the initial search, a second search was conducted including the following search terms: ‘group model building’ OR ‘participatory dynamic simulation model’ OR ‘participatory dynamic simulation modeling’ OR ‘participatory model’ OR ‘participatory modeling’ yielding 1235 documents. VL screened and sorted records, initially by reviewing titles and abstracts and then by reviewing full papers. Foundational papers in the literature were supplemented with a focus on more recent publications between 2017 and 2021, to capture current thinking and innovations around participatory methods. This preliminary search was aided by citation tracking and snowballing, as well as recommendations from LF and JO, both with extensive experience in participatory modelling.

Reflecting on these searches, the authors iteratively tailored the search strategy toward literature with a specific emphasis on:

- Models aimed at forecasting future trajectories and engagement with policy questions and decisions
- Transparency, socialisation and communication of models
- Novel methods for integrating data/knowledge and bringing diverse groups of people together around the development and use of models

This search led to a total of 53 texts being included for review, which are summarised below in Table 1.

**Table 1:** Summary of texts included in the review

Author and year	Type of text	Main discipline	Main focus
Adams et al. [25] (2021)	Review/theoretical	Health policy, science and technology studies	Explores participatory modelling literature within health and in water management; draws on STS theory to think about new approaches for health modelling
Anderson [11] (2021)	Theoretical	History of public health COVID modelling	Examines modelling as a “crisis technology” – considers how COVID might be known as a more heterogenous object of knowledge
Atkinson and Wells [79] (2015)	Review/theoretical	Health policy	Reviews literature to determine range of uses of system dynamics modelling for health policy, and the effectiveness of these applications
Atkinson and Knowles [58] (2018)	Case study	Health policy—alcohol policy	Explores feasibility of participatory agent-based models to support decision-makers and stakeholders test different policy scenarios in context of complexity and uncertainty around alcohol policy decisions
Atkinson and Skinner [32] (2020)	Position paper/Case study	Public health; mental health policy	Discusses need for a predictive planning framework for mental health policy and the role of systems models and simulation to inform decision making
Atkinson and Skinner [36] (2020)	Case study	Mental health policy	Describes the development of a participatory system dynamics model and its use in predicting trends in suicidal behaviour based on different intervention scenarios
Black and Andersen [68] (2012)	Theoretical/Methodological	Systems science	Discusses models as sociological boundary objects; deals with friction in collaboration
Bou Nassar and Malard [56] (2021)	Case study	Water management	Explores storytelling techniques as part of participatory approach to modelling with marginalised, linguistically diverse indigenous communities in Mayan Guatemala
Brugnach and Tagg [43] (2007)	Case study	Water management	Explores policymakers’ lack of trust in modelling tools; shows pubic confidence models is dependent on addressing uncertainties
Callon and Law [74] (2005)	Theoretical	Sociology	Explores the complex nature of the boundary between qualitative (judgement) and quantitative (calculation). Develops concept of ‘qualculation’ to capture the judgement/passion aspect of number-based reasoning
de Oliveira Morais and Kuhlberg [46] (2021)	Case study	Health policy and community-based system dynamics	Examines impact of modelling exercises on knowledge to policy translation
Deutsch and Lustfield [50] (2021)	Case study	Public Health	Explores method to increase role of ‘personal experience’ participants in model building
Elsawah and Filatova [24] (2020)	Review/Theoretical	SES modelling	Outlines key challenges for modelling: including ‘bridging epistemologies across disciplines’, combining quant/qual methods and data sources, ‘integrating the human dimension’
Falconi and Palmer [60] (2017)	Theoretical/Methodological	Water resource management	Develops 2-stage evaluation framework to show effective participatory models (1) facilitate dialogue (as boundary objects) and (2) improve accessibility of technical knowledge
Freebairn and Rychetnik [44] (2017)	Empirical—3 case studies	Health policy	Describes how systems science methods can build on knowledge mobilisation approaches for public health topics
Freebairn and Atkinson [45] (2018)	Empirical—3 case studies	Health policy	Explores experience of model building participants, including policymakers and health service providers and their perceptions of value and efficacy of modelling
Freebairn [39] (2019)	Case Study	Health policy	Doctoral thesis exploring implementation and value of participatory systems modelling of Diabetes in Pregnancy in Australia
Freebairn and Atkinson [42] (2019)	Empirical—3 case studies	Health policy	Explores decision-making processes in participatory model development for public health policy practice
Frerichs and Lich [62] (2016)	Case study	Community health	Explores how application of social theory can constructively aid group modelling process for health insights
Gilbert and Terna [34] (2000)	Theoretical/Methodological	Computer simulation/Social science	Describes techniques for building agent-based models as a ‘third way’ of doing social science
Gray and Gray [64] (2013)	Theoretical/Methodological	Systems science	Describes design and use of participatory modelling tool ‘Mental Modeler’ that surfaces mental models of stakeholders
Hosseinichimeh and Kim [48] (2019)	Case study	Systems science	Explores how different levels of stakeholder behaviour—individual, organisational and policy—are reflected in model building
Huang, Hmelo-Silver [65] (2018)	Case study	Citizen science	Explores how ‘Mental Modeler’ participatory modelling tools functions as a boundary object in citizen science projects
Ibrahim Shire and Jun [33] (2020)	Case study	Healthcare systems	Examines perspectives of health care workers as participants in system dynamics modelling
Johnson [22] (2015)	Case study	Water and Soil Management	Explores role of ABMs as ‘interested amateurs’ to help draw out discussions among policy experts
Jordan and Gray [23] (2018)	Review article	SES modelling	Presents key questions to guide future participatory modelling inquiry
Kaehne [2] (2021)	Theoretical/Methodological	Modelling and COVID evaluation	Response to Pawson [1]—Epidemiological modelling in COVID has shown the dangers of neglecting local conditions by making assumptions about generalised living conditions of communities
Lahsen [73] (2005)	Case study	Social studies of science	Draws on ethnography with climate scientists to explore the epistemology of models and how uncertainties operate as part of modelling practice
Landström et al. [26] (2011)	Case study	Water management, social studies of science	Examines public controversy and participatory modelling experiment in water management to explore the contribution of non-scientific expertise to environmental knowledge
Langellier and Kuhlberg [37] (2019)	Case study	Health policy and community-based system dynamics	Explores role of participatory modelling in 10 Latin American countries for engaging stakeholders in complex systems thinking
Matthews and Gilbert [40] (2007)	Review article	Land-use modelling	Reviews applications of agent-based land-use modelling to understand the value of the tools as decision support tools
Muttalib and Ballard [49] (2021)	Case study	Healthcare systems	Explores role of group model building in resource limited setting to create shared mental model in health services
O’Donnell and Atkinson [35] (2017)	Theoretical	Health policy	Explores how models engage a range of evidence typologies. Emphasises that complex problems require integration and triangulation of a range of evidence types
Occhipinti and Skinner [38] (2021)	Case Study	Mental health policy	Explores how systems modelling can mitigate competing priorities between different levels of government that undermine investment
Osgood [41] (2017)	Theoretical	Systems science/public health	Reflects on trends in health applications of systems science and the implications for health modelling
Østebø [12] (2021)	Monologue/Case study	Anthropology	Explores how a village in Ethiopia has become a policy model. Considers how policy models arise, how they travel and how the village’s status is impacted by being a model
Pawson [1] (2021)	Theoretical/Methodological	Modelling and COVID evaluation studies	Calls for evaluation methods to be applied to models
Rhodes and Lancaster [80] (2020)	Theoretical	Public health sociology	Describes how models are not only informing evidence-making policy decisions but they are feeding citizen science and social actions around COVID
Rhodes and Lancaster [81] (2020)	Theoretical	Public health sociology	Explores public controversies around COVID models and considers the implications of a science-in-action approach to modelling which explores the social life of models
Rhodes and Lancaster [75] (2020)	Case study	Public health sociology	Explores how pandemic models relate to implementation contexts and the implications for evidence-based approaches in global health
Rhodes and Lancaster [72] (2021)	Empirical study	Public health sociology	Builds on Callon and Law [74] to explore how model results travel and go beyond ‘evidence-based’ ideas when they move in policy and practice spaces
Rouwette and Bleijenbergh [63] (2016)	Case study	Public policy	Explores modelling practice in ambiguous and conflicted situation
Rouwette and Korzilius [61] (2011)	Empirical study	Social psychology and systems science	Explores role of group model building in changing attitudes, subjective norms and intentions. Counterintuitive insights are crucial but participants don’t often recognise mental model changes
Saltelli and Bammer [82] (2020)	Theoretical/Best practice guide	Modelling and COVID	Outlines ‘best practices’ for responsible, transparent modelling including ‘match purpose with context’ as results from model will reflect the interests, disciplines and biases of the developers
Schubert [70] (2015)	Theoretical	Science and technology studies	Examines how computer simulations generate new forms of social relations
Singer and Gray [66] (2017)	Case study	Environmental science	Explores how using ‘Mental Modeler’ tools in participatory modelling in crisis can help community recovery efforts
Siokou and Morgan [47] (2014)	Review article	Health policy and practice	Discusses evidence of effectiveness of group model building approaches applied in preventive health
Smetschka and Gaube [57] (2020)	Case study	Agricultural development	Discusses potential impact of participatory modelling for transdisciplinary research in relation to 3 types of knowledge: systems, target and transformation knowledge
Sterling and Zellner [53] (2019)	Review article	Environmental modelling	Draws on reflections of experienced modellers and review of literature to explore how participatory modelling builds collective knowledge and social capital
Van Bruggen and Nikolic [55] (2019)	Review/theoretical	Climate policy	Explores the role of models in wider system transformation. Distinguishes transformative approaches as those that engage in process of learning and critical reflection of problems
Voinov and Kolagani [52] (2016)	Review article	Environmental modelling	Reviews principles that guide participatory modelling and how stakeholder participation is changing with new technology advances and methods
Voinov and Gaddis [59] (2017)	Theoretical/methodological	Environmental modelling	Argues that modellers embrace their values. Modellers have responsibility to communicate model results for public understanding and are framed to influence values in an appropriate way
Williams [54] (2020)	Theoretical	Climate change adaptation	Describes necessary conditions for participatory modelling for climate change adaptation to increase autonomy of marginalised stakeholder groups

The texts were read closely for key concepts describing the purpose of models or engagements with stakeholders. We focused on instances in the texts where the logic of value was invoked and where value was described in relation to the development and communication of simulation models. The epistemologies, methods and focus of the texts are summarised in Table 1. As the table indicates, these papers comprised a mixture of case studies, review articles and theoretical papers. In keeping with a hermeneutic approach, the analysis involved a dialectical tacking back and forth between descriptive detail and the broader themes in the literature [28]. This allowed for new ways of linking concepts and synthesising theories. Themes were identified by VL and presented to LF and JO for feedback where we addressed contradictions and refined the descriptive labels and statements. This approach conforms to Standards for Reporting Qualitative Research [31] (Additional file 1).

## Results

**Fig. 1:**
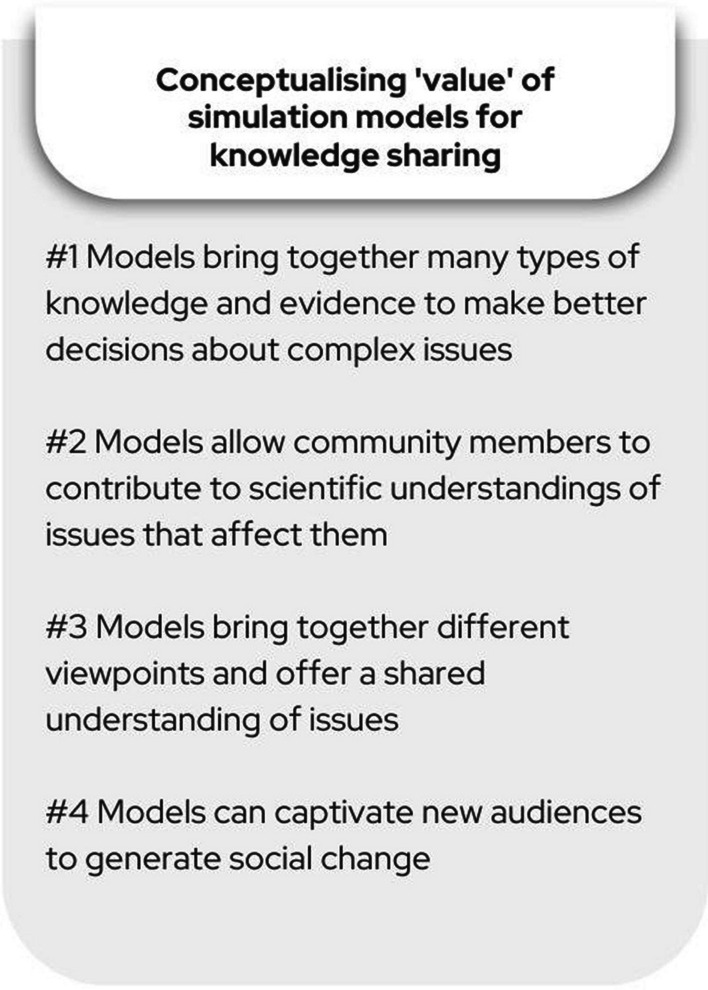
Summary of narrative conceptions

Results are presented as four over-arching narrative conceptions of the role and value of models (see Fig. 1). We identified cross-cutting themes around approaches to participation, knowledge sharing and public engagement. These narrative conceptions are not mutually exclusive thematic domains, but are in some cases overlapping and in conversation with one another.

### Narrative conception #1: models simulate and help solve complex problems

Models in this research tradition are often referred to as ‘decision-support’ tools that seek to provide predictive planning frameworks to inform and improve policy decisions [32]. In this conceptualisation, models are designed to help policymakers gain better understandings of system behaviour and the multiple challenges and goals in a complex system [33]. The kinds of decisions that models in this tradition are supportive of include service planning, investment strategies, and policy reform. Models act to facilitate sophisticated and intricate understandings of a problem and how it will respond to alternative courses of action, allowing decision-makers to identify more effective intervention strategies [34]. Through forecasting implications of particular investment strategies models have the potential to both optimise resource allocation and minimise unanticipated harmful consequences of decisions.

This conceptualisation of models often draws on the modelling methods of complex systems science (including system dynamics modelling), which examine interrelationships, feedback loops and the presence of equilibria in the system under investigation. The aim of such modelling is to move beyond simple ‘rational’ choice decisions, based on straightforward inputs and outputs, in order to identify opportunities for intervention or action that might have been overlooked by traditional ‘evidence-based’ or cost/benefit approaches. The inclusion of local and practice-based knowledge alongside research data is considered to challenge accepted conventions of evidence ‘hierarchies’ in health [35]. Further, concepts of evidence and data are not understood in terms of isolated individual studies, but rather as a ‘complex puzzle’ which takes account of the additive effects of combining different intervention approaches over time [36]. Modelling can be undertaken at different levels of abstraction (i.e., micro-, meso-, macro-) to arrive at a testable hypothesis and explanation for why a system behaves the way it does [37]. Systems models therefore allow decision-makers to consider how competing priorities and agendas across, for example, different levels of government may undermine the impact of desired policy outcomes [38]. Agent Based Modelling in particular is favoured by those interested in the activities and/or heterogeneity of individual agents (such as people, equipment, services or vehicles) [39]. These models strive toward a depiction of detailed reality through the inclusion of individual behaviour, social interactions and dynamics, and environmental variations [40].

Approaches to participation of stakeholders and public engagement in this tradition are varied. In the participatory development of Agent Based Models, experts are described as being able to easily relate to the depiction of the model at an individual level as they can draw on experiential knowledge of patient case histories and deploy professional judgement to validate and help calibrate the model [41, 42]. This and other approaches are underpinned by an ideal of ‘transparency,’ which eschews the ‘black box’ model in favour of making the model structure, assumptions, and limitations available for outside appraisal to assist in interpreting model results [43]. Others draw on the concept of ‘knowledge mobilisation’ aiming to include ‘end users’ (i.e. decision-makers) of the model in the model development process [32, 44]. Stakeholders are typically policymakers, researchers and practice experts (e.g. clinicians) whose participation is said to increase trust in the model outputs [45], ensure consideration of practical issues with policy implementation or evaluation [46] as well as the translatability of the model findings [35].

Benefits of participation in this tradition include opportunities for capacity building in terms of knowledge and expertise in systems science and modelling methods [37, 47]. This expertise is theorised to improve literacy in interpreting modelling results and empower end-users make better appraisals of future models they encounter in their policy practice [37]. Participatory modelling can be used to aid planning and policy choices in terms of organisational management, to enhance learning and promote shared understanding of complex problems among different actors in the system [33, 48, 49].

Questions surrounding language, accessibility, cultural adaptation, and power dynamics in the modelling process and translation of model results are rarely explored in depth in this approach. Some have pointed out that recruiting a diversity of participants and stakeholders in this tradition tends to favour experts such as health practitioners or policymakers. By contrast, small numbers of community participants are chosen to represent a variety of personal experiences within the system as a whole [50].

### Narrative conception #2: models as tools for community engagement

Drawing on principles of community-based participatory research, community development and equity, the emphasis in this conceptualisation is on modelling practice as a ‘powerful learning process’ [23]. Research in this tradition is in keeping with efforts to engage in dialogues and enhance communication between experts and lay publics [51]. Drawing on trends in citizen science, it responds to demands by citizens to be included as stakeholders in planning decisions that affect them [52].

Central to this narrative construct is a focus on harnessing the capacity of multiple publics to engage in coordinated efforts to address the problem/s at hand. Goals of social learning and building ‘social capital’ among participants take priority over collectively producing model results [53]. This orientation is often underscored by a logic that the ‘problems’ tackled through modelling disproportionately affect marginalised groups, therefore meaningful participation of such groups is critical to the success of modelling efforts. Community-based participants are treated as experts of local and historical context that bring crucial knowledge, skills and labour to the model building [52, 53]. Such groups may be identified by virtue of their experience—for example people with personal experience of substance abuse and domestic violence [50], or by virtue of their relationship to place and experience of historic initiatives (e.g. First Nations people). Concepts of evidence and data are construed as contingent in this tradition and there is a recognition that vast diversity of participant experiences exists in relation to modelling issues of interest [50]. Knowledge produced through modelling exercises is therefore fluid and mediated by an “interactive and iterative” learning process [54].

Commitment to engaging a broad range of community participants is not only practical, but also ideological [55], encouraging modelling practice to strive toward engaging participants in ways that empower them to take ownership of the products of the modelling [52]. Power dynamics—and who may be left out of participatory modelling—are of key concern within this research tradition, and authors emphasise how powerful stakeholders can encourage, or conversely prevent, other actors from fully participating [53]. Attending to unequal power dynamics is seen as essential for encouraging engagement of marginalised community members, and methodological approaches carefully consider how to meaningfully engage all participants and ‘amplify’ the voices of previously marginalised stakeholders in the modelling process [54, 56]. Many approaches to modelling in this tradition experiment with forms of knowledge production that go beyond scientific publication, often using arts and performance-based mediums such as storylines, blogs, and alternative forms of visualisation [53, 54]. In addition to experimenting with communication mediums, many authors also draw attention to the issue that some marginalised participant groups may lack the necessary resources (time, financial, psychological, transportation) to take part in projects requiring intensive commitment [50], calling for compensation or remuneration to remedy this. Many authors stress, however, that communication of model results should not be confined to the model builders and decision makers. An enduring question in this tradition is how stakeholders who participated in the model building can take their new understandings and communicate them to others who were not involved in the model construction [57].

### Narrative conception #3: models as tools for consensus building

This conceptualisation of models builds on and overlaps with narrative conceptions #1 and #2, but is distinguished by its emphasis on the value of modelling processes for facilitating communication and providing opportunities for negotiating conflicts and building consensus among stakeholder groups. Much of the research on participatory modelling practice acknowledges the value of modelling practice for promoting intellectual exchange and advancing contentious debates. Models are described as platforms for strengthening relationships between different knowledge communities (e.g. academics and policymakers [58] or historically antagonistic institutions [59]). Some texts take this concept further, delving deeply into the role of models and the specific qualities inherent in modelling that lends this practice to consensus building.

Approaches to participation in this tradition are centred on the negotiation of conflict, or reconciling different viewpoints among participants. A major impetus for modelling in this conception is the development of a ‘shared language’ among participants [60]. This is important because modelling is often used to address complex and messy problems and participants bring with them rich, but often partial, prior knowledge of the situation [61]. Participants developing a ‘shared understanding’ of the issue being modelled—be it policy implementation or the overview of a system—is seen as an inherently desirable outcome of the modelling process [46].

Issues of bias and values are also of central concern. Tools and methodologies are deployed to elicit unconscious bias [62] and recognise beliefs and values [23]. Voinov and Gaddis [59] have been particularly influential in arguing that modellers ought to acknowledge and embrace how their work is driven by ‘values’. Others have drawn on psychological theories of persuasion and mental model change to explore how model building processes influence beliefs, attitudes and perceptions of norms [61]. This research identifies “counter-intuitive insights”—where model simulations or views of other participants run counter to originally held positions on an issue—as a key strength in shifting mental models [63]. Mental modelling techniques and tools have been popularised by the work of Gray and colleagues [64] to facilitate engagement and communication between participants [65] and track changes in mental models during model building [66].

‘Boundary object’ theory is often invoked in this research tradition. Many of these texts draw on Star and Greisemer’s [67] theoretical synthesis of Boundary Objects in Science and Technology Studies. Models are depicted as ‘adaptable’ objects whose flexible representation enables people with different backgrounds and expertise to communicate more effectively and engage in co-ordinated activities [65]. Texts emphasise and analyse models’ ability to facilitate communication on contentious issues through developing a shared language and a shared understanding between different groups [68]. From this perspective, what matters is less a model’s precision than its capacity to provide a platform that can mitigate conflict and friction between different groups.

### Narrative conception #4: models as volatile technologies that generate social effects

This conceptualisation of models is inspired by efforts from the social sciences to critique dominant approaches to modelling. In contrast to the other three narrative conceptions, this approach is less concerned with developing ‘best practice’ approaches (themselves premised on another kind of model) than with rethinking taken-for-granted assumptions within modelling.

Science and Technology studies, and the work of Bruno Latour [69] has a strong influence on studies in this tradition. In this sense, models are not simply neutral technologies that describe or represent activities and issues of policy interest, they are “generative” [26] and “transformational” [70]. Models—like other products of science in Latour’s theorisation—are considered for their ability to generate social effects. They are dynamic entities or “assemblages,” clusters of actors, practices, discourses and material objects which circulate across social fields and intersect with policy practice. Models and modelled evidence are multiple in their meanings and are perpetually emerging. This conceptualisation of models may also be framed within broader critical approaches to anticipatory governance [71], New Public Management [12] and practices of ‘projection’ or ‘simulation’ [72, 73].

Callon and Law’s [74] conceptual reconfiguration of the concept of calculation to include notions of judgement and passion has spawned a number of texts exploring the ‘affective’ and emotional components of modelling practice [72]. The affective qualities of models, and their ability to generate and be shaped by emotional responses, have led some authors to note the “virality” of models and their virus-like capacity for contagion [12]. Some suggest that the ‘seductive simulations’ tempt modellers to ‘oversell’ their products as ‘truth machines’ [73]. Such studies emphasise the messiness and uncertainties involved in modelling practice.

Knowledge and ‘evidence’ are characterised in this tradition as tentative, evolving, contingent and emergent, highlighting the limits of conceptualisations of models based on ‘evidence-based’ approaches. Some, such as Pawson [1], draw on complexity science and evaluation theory to question the evidence that informs health modelling. This critique further extends to dominant conceptions of ‘interventions’ as the evidence upon which model assumptions are based, stressing “the impossibility, of trying to capture a complex, self-transforming process as a model ‘parameter’” [1]. Texts highlight the dangers of building models based on evidence from interventions which are regarded as ‘fixed’ both in terms of implementation and effect. The focus here invokes questions around the role of “context” in modelling practice. Whilst acknowledging that some models do make generalised assumptions while paying insufficient attention to local conditions, this perspectival view (unlike Narrative conception #2) does not pursue a line of reasoning whereby better models depend on increasingly attuning them to local conditions [2, 75]. Models, after all, will always be “simplifications based on abstraction” [2]. Instead, both interventions and models are thought of as fluid and continually adapting as elements of ‘evidence-making interventions’ [76]. A model may function as an ‘interested amateur,’ adopting the role of an outsider that aids the interaction of policy experts, freeing up participants to openly voice criticisms and confront sensitive issues [22]. In this way, a measure of a good model is not simply how well it incorporates local data within its calculations, but how well models are mobilised as part of an adaptive science in which they can be treated as a pathway to dialogue, and deeper appreciation of interventions and their effects.

## Discussion

Other reviews of participatory modelling have explored the value of stakeholder engagement in a particular field (e.g. social and environmental sciences or health). This review, however, examines the narrative conceptualisations of the value of models across a range of fields that engage with participatory modelling approaches. Examining these conceptions side-by-side allows us to consider how learnings from different fields might inform one another. In each of these narrative conceptions, value is constituted through the *process* of building models with stakeholders. In narrative conception 1, models employ quantitative methods but are embedded in social processes that are pivotal to their success or failure. The process of participatory modelling enhances the quality of model results by integrating new forms of knowledge and building trust with decision-makers. In other conceptions, the process is less about refining model accuracy and more about empowering marginalised stakeholders (narrative conception 2) or negotiating friction and developing a shared language among model participants from different knowledge communities (narrative conception 3). In narrative conception 4, the process is characterised as less goal-oriented, instead describing the unpredictability, multiplicity and self-organisational properties of model building with stakeholders.

An important point of difference in these conceptualisations lies in whether models are considered to be analytic tools to support decision-making, or whether modelling is itself a form of intervention capable of generating social change. Different framings of the role and value of modelling have implications for the evaluation of modelling and understanding the effects of a modelling project. Evaluations of participatory modelling are often underdeveloped and there is a need to develop clearer appreciation of the critical elements entailed in the purpose and associated value of modelling tools [77, 78]. If models are framed as tools for supporting decision-making (narrative conception 1), evaluation would focus on investigating the experience of participant stakeholders including their understanding and perception of the problem, or their trust in the legitimacy of model results [60, 79]. If models are deployed as aides in collaborative problem-solving, or as conflict resolution tools (narrative conception 3) evaluation might focus on how model representations are received among the group, along with their capacity to de-personalise conflicts and allow participants to negotiate less threatening paths to develop shared language [63] and shape consensus [68]. If models are avenues for building social capital or empowering traditionally marginalised communities (narrative conception #2), then evaluation of conflict resolution, or assessments of the development of new knowledge among participants must also attune to attenuating power dynamics and the foregrounding of marginalised or non-scientific forms of knowledge. Importantly, the goal of empowerment ought not to focus on individual participants, but facilitating circumstances so that publics are capable of producing new knowledge [25]. In narrative conception #4, modelling is framed as a starting point or a trigger for generating change processes. This has ramifications for evaluation in that the role of the model can be considered at project initiation, however flexibility is needed as the role may change over time and with different audiences and contexts.

These narrative conceptions may alternatively be used as a package to aid modellers in communicating the role and value of modelling tools to stakeholders including policy experts, funders, media or community representatives. Drawing on all four narrative conceptions of value can help illustrate how models serve multiple purposes and broaden understandings of their role in change processes. If the value of models is too narrowly defined, if their predictive value is overstated, or based on notions of infallible evidence to predict outcomes of policy decisions, there is a risk that the impact of modelling tools may be overlooked.

## Conclusions

Crises such as the COVID pandemic and environmental catastrophes offer opportunities to transform the dynamics of knowledge sharing and engagement with models across diverse community and policymaking contexts. Bringing together the different narrative conceptions highlights the *multiple* ways that simulation modelling can be of value for public policy engagement. Linking ideas in these narrative conceptions—for example, considering how model representations can sit alongside and speak to non-scientific ways of knowing in informing policy decisions—offers new possibilities for harnessing the value of modelling. It may also prompt critical reflection on taken-for-granted ideas such as the value of consensus. The development of consensus or shared language must be weighed against opportunities for building social capital or encouraging participants to better articulate and communicate alternative perspectives and ways of understanding issues. In this sense, modelling may be considered valuable for its potential to generate and legitimate multiplicity.

At the same time, if the value of modelling is also located in the process of model-building or collaborative interaction with models, more work is needed to understand the evolutionary dynamics and systemic transformations that are potentially triggered by modelling beyond the participants directly engaged in model development. Future research tracking the value of models and their ripple effects as they circulate in wider public and policy spheres is needed. These different narrative conceptions may be used as a starting point for understanding the conditions under which simulation modelling can generate proactive pathways for change, or alternatively risk perpetuating the status quo.

### Supplementary Information

**Additional file 1: **Standards for reporting qualitative research.

## Data Availability

Not applicable.
