# Supplementary material for: Conceptualising the value of simulation modelling for public engagement with policy: a critical literature review
Source: Health Res Policy Syst. 2023 Nov 27;21:123. doi: 10.1186/s12961-023-01069-4 (PMC10680332; doi:10.1186/s12961-023-01069-4)
Supplement: Supplementary file 1 — Additional file 1: Standards for reporting qualitative research. [file 12961_2023_1069_MOESM1_ESM.docx]

**Appendix 1. Standards for Reporting Qualitative Research**

|  | **Standards for Reporting Qualitative Research (SRQR)*** |  |
| --- | --- | --- |
|  | <http://www.equator-network.org/reporting-guidelines/srqr/> |  |
|  |  |  |
| **Title and abstract** | |  |
|  | **Title** - Concise description of the nature and topic of the study Identifying the study as qualitative or indicating the approach (e.g., ethnography, grounded theory) or data collection methods (e.g., interview, focus group) is recommended | The title specifies that we have conducted a “critical literature review” (p1) |
|  | **Abstract** - Summary of key elements of the study using the abstract format of the intended publication; typically includes background, purpose, methods, results, and conclusions | The abstract (p2) provides a summary of the key elements in an unstructured format |
|  |  |  |
| **Introduction** | |  |
|  | **Problem formulation** - Description and significance of the problem/phenomenon studied; review of relevant theory and empirical work; problem statement | We have described the significance of the research by drawing on recent literature discussing shifts in modeling during the COVID pandemic (p3-4) and relevant theory regarding social dynamics of modeling practice (p4-5). The problem statement describes how current literature is restricted to particular sectors and the value models hold in wider societal contexts is unclear (p5). |
|  | **Purpose or research questio**n - Purpose of the study and specific objectives or questions | The purpose of the review was to develop a better understanding of how ‘value’ of modeling is conceptualized in different research traditions and to develop a more coherent conception of the multiple conceptions of the value of modeling that exist across different sectors. |
|  |  |  |
| **Methods** | |  |
|  | **Qualitative approach and research paradigm** - Qualitative approach (e.g., ethnography, grounded theory, case study, phenomenology, narrative research) and guiding theory if appropriate; identifying the research paradigm (e.g., postpositivist, constructivist/ interpretivist) is also recommended; rationale** | The review employs hermeneutics as a philosophy and method for analysis and synthesis of learnings which is in keeping with an interpretivist research paradigm. In keeping with Critical Literature Review methodology, this approach emphasizes induction, interpretation and critique of narrative descriptions in the literature to develop a more sophisticated understanding of the concept of value. (p6)  We have chosen this method over a Systematic Review method because we were interested in storylines in the literature rather than answering questions of effectiveness or ‘what works’ (see p6-7). |
|  | **Researcher characteristics and reflexivity** - Researchers’ characteristics that may influence the research, including personal attributes, qualifications/experience, relationship with participants, assumptions, and/or presuppositions; potential or actual interaction between researchers’ characteristics and the research questions, approach, methods, results, and/or transferability | VL has extensive experience in qualitative research with an emphasis on the social studies of science and technology, public health and policy research. This enabled VL to review the literature using an ‘outsider’ lens to formulate novel approaches to synthesizing the literature. LF and JO both have extensive experience in participatory modeling. The review drew on their expertise both to recommend key literature, as well as to validate the findings from the review in relation to their own experiences of modeling practice. |
|  | **Context** - Setting/site and salient contextual factors; rationale** | The context of the review is simulation modelling for public engagement with policy – we have focused on this context due to the increasing influence of such models in both policy and public spheres, particularly in relation to the climate and COVID crises. |
|  | **Sampling strategy** - How and why research participants, documents, or events were selected; criteria for deciding when no further sampling was necessary (e.g., sampling saturation); rationale** | Documents were selected for review based on SCOPUS searches using search terms described on p7 followed by discussions and analysis with the research team to iteratively refine the focus of the literature review. Inclusion criteria: English language, scholarly documents between 2006-2021. Citation tracking and discussions with authors as well as other modelling experts, peer reviewers and social science researchers studying models also led to inclusion of additional sources and led to refined focus on foundational literature and sources between 2017 and 2021. No further sampling was necessary when saturation of themes was identified. |
|  | **Ethical issues pertaining to human subjects** - Documentation of approval by an appropriate ethics review board and participant consent, or explanation for lack thereof; other confidentiality and data security issues | N/A – literature review only |
|  | **Data collection methods** - Types of data collected; details of data collection procedures including (as appropriate) start and stop dates of data collection and analysis, iterative process, triangulation of sources/methods, and modification of procedures in response to evolving study findings; rationale** | See sampling strategy |
|  | **Data collection instruments and technologies** - Description of instruments (e.g., interview guides, questionnaires) and devices (e.g., audio recorders) used for data collection; if/how the instrument(s) changed over the course of the study | See sampling strategy |
|  | **Units of study** - Number and relevant characteristics of participants, documents, or events included in the study; level of participation (could be reported in results) | 53 documents selected for review based on emphasis in key areas of interest: models aimed at forecasting and policy; transparency and communication of models; methods for bringing diverse groups to collaborate around models (p8) |
|  | **Data processing** - Methods for processing data prior to and during analysis, including transcription, data entry, data management and security, verification of data integrity, data coding, and anonymization/de-identification of excerpts | Data processing was an iterative process of VL screening and sorting based on document abstracts and tabulation of a summary of sources in Word. Papers of potential relevance were then reviewed in full and coded using highlights and notes in Word to help develop themes. |
|  | **Data analysis** - Process by which inferences, themes, etc., were identified and developed, including the researchers involved in data analysis; usually references a specific paradigm or approach; rationale** | Initial themes were refined through iterative discussions and reflective theme development with the entire authorship group. |
|  | **Techniques to enhance trustworthiness** - Techniques to enhance trustworthiness and credibility of data analysis (e.g., member checking, audit trail, triangulation); rationale** | Peer review process, international conference presentations and discussions with numerous modeling experts have indicated that the themes resonate with modelling practice and experience of other social scientists studying modeling. |
|  |  |  |
| **Results/findings** | |  |
|  | **Synthesis and interpretation** - Main findings (e.g., interpretations, inferences, and themes); might include development of a theory or model, or integration with prior research or theory | The main findings are summarized in a Conceptual model of four narrative conceptions of the ‘value’ of simulation models for knowledge sharing. See Figure 1 on p9 |
|  | **Links to empirical data** - Evidence (e.g., quotes, field notes, text excerpts, photographs) to substantiate analytic findings | The findings are substantiated through citations of different sources throughout the results section. |
|  |  |  |
| **Discussion** | |  |
|  | **Integration with prior work, implications, transferability, and contribution(s) to the field -** Short summary of main findings; explanation of how findings and conclusions connect to, support, elaborate on, or challenge conclusions of earlier scholarship; discussion of scope of application/generalizability; identification of unique contribution(s) to scholarship in a discipline or field | The findings articulate four narrative conceptualisations of value of modelling: 1) models simulate and help solve complex problems; 2) models as tools for community engagement; 3) models as tools for consensus building; 4) models as volatile technologies that generate social effects. We discuss how these ideas of ‘value’ overlap and what they offer one another. We go on to connect this to literature on evaluation of participatory modelling and how the findings can be used to communicate value of modelling to diverse audiences. |
|  | **Limitations** - Trustworthiness and limitations of findings | The findings are only a starting point and more work is needed a) to understand system-wide transformations b) to explore effects of modelling beyond core participants. Future research on value of models in wider public and policy spheres is recommended. |
|  |  |  |
| **Other** | |  |
|  | **Conflicts of interest** - Potential sources of influence or perceived influence on study conduct and conclusions; how these were managed | None declared p21 |
|  | **Funding** - Sources of funding and other support; role of funders in data collection, interpretation, and reporting | p21 |
|  |  |  |
|  | *The authors created the SRQR by searching the literature to identify guidelines, reporting standards, and critical appraisal criteria for qualitative research; reviewing the reference lists of retrieved sources; and contacting experts to gain feedback. The SRQR aims to improve the transparency of all aspects of qualitative research by providing clear standards for reporting qualitative research. |  |
|  |  |  |
|  | **The rationale should briefly discuss the justification for choosing that theory, approach, method, or technique rather than other options available, the assumptions and limitations implicit in those choices, and how those choices influence study conclusions and transferability. As appropriate, the rationale for several items might be discussed together. |  |
|  |  |  |
|  | **Reference:** |  |
|  | O'Brien BC, Harris IB, Beckman TJ, Reed DA, Cook DA. **Standards for reporting qualitative research: a synthesis of recommendations.** *Academic Medicine*, Vol. 89, No. 9 / Sept 2014  DOI: 10.1097/ACM.0000000000000388 |  |
